# Olorofim activity against multidrug-resistant *Fusarium* unveils intra-species and inter-species variability

**DOI:** 10.1128/aac.00961-25

**Published:** 2025-10-10

**Authors:** Ruoning Xue, Shenghan Gao, Sybren de Hoog, Anne van Diepeningen, Shaoqin Zhou, Tianyi Xu, Like Fokkens, Ruoyu Li, Lin Bai, Zhe Wan, Paul E. Verweij, Yinggai Song

**Affiliations:** 1 Department of Dermatology and Venerology, Peking University First Hospital 26447, Beijing, China; 2 Research Center for Medical Mycology, Peking University 12465, Beijing, China; 3 National Clinical Research Center for Skin and Immune Diseases, Beijing, China; 4 Institute of Microbiology, Chinese Academy of Sciences 85387, Beijing, China; 5 Department of Medical Microbiology, Radboud University Medical Center 6034, Nijmegen, the Netherlands; 6 Radboudumc-CWZ Center of Expertise for Mycology, Radboud University Medical Center 6034, Nijmegen, the Netherlands; 7 Foundation Atlas of Clinical Fungi, Hilversum, the Netherlands; 8 BU Biointeractions and Plant Health, Wageningen Plant Research, Wageningen University and Research 4508, Wageningen, the Netherlands; 9 Laboratory of Phytopathology, Plant Sciences Group, Wageningen University and Research 4508, Wageningen, the Netherlands; 10 Department of Biophysics, School of Basic Medical Sciences, Peking University 208325, Beijing, China; 11 Center for Infectious Disease Research, Diagnostics and Laboratory Surveillance, National Institute for Public Health and the Environment (RIVM) 10206, Bilthoven, the Netherlands; University of Iowa, Iowa City, Iowa, USA

**Keywords:** susceptibility, *Fusarium*, olorofim

## Abstract

Fungi in the genus *Fusarium* are plant pathogens but are also capable of causing a wide range of diseases in humans. The intrinsic multi-drug resistance of *Fusarium* often leads to a poor clinical outcome in patients with severe immune disorders. Olorofim, a member of the orotomide class, is a novel type of antifungal drug that interferes with pyrimidine biosynthesis by inhibiting dihydroorotate dehydrogenase and thereby prevents growth and cell division. In this study, the *in vitro* activity of olorofim was evaluated against 253 *Fusarium* isolates, of which 228 isolates belonging to the prevalent complexes involved in human infection, *F. solani* species complex (SC) and *F. fujikuroi* SC. All *Fusarium* isolates underwent species-level identification via multi-locus sequence typing (MLST) targeting *RPB1*, *RPB2*, and *TEF1* loci. Antifungal susceptibility testing was performed using a CLSI M38, 3rd ed. broth microdilution for olorofim. The geometric mean of the MICs of olorofim for all 253 isolates was 0.581 µg/mL, ranging from 0.015 µg/mL to >16 µg/mL. Olorofim demonstrated high MICs against *F. solani* SC, whereas greater potency (lower MICs) was observed against *F. fujikuroi* SC. Clinical isolates tended to have higher MIC values than environmental isolates, but this pattern was not consistent across all species complexes. Overall, olorofim demonstrated moderate *in vitro* activity against *Fusarium* isolates, suggesting it might be a potential candidate for treating fusarioses caused by multidrug-resistant strains.

## INTRODUCTION

Fungi of the genus *Fusarium* are universally found in the environment and are among the major plant pathogens. In addition, human (and animal) infections are frequent in some regions of the world causing a wide spectrum of diseases, ranging from mild nail infections, skin infections, and traumatic keratitis (1–4), to fatal dissemination in immunocompromised individuals (5, 6). Invasive infections are observed in patients with profound neutropenia or severe T-cell immunodeficiency and are particularly recalcitrant to antifungal therapy. Currently, approximately 30 *Fusarium*-like species have been documented to be involved in human infection (7), but depending on species concepts maintained, where species are classified as clinically relevant pathotypes rather than taxonomic species complexes, some authors claim that this number will grow (8). Isolates most frequently encountered in human infection belong to the *Fusarium solani* species complex (SC) (FSSC; = *Neocosmospora*) and the *F. oxysporum* SC (FOSC) (2, 9).

Clinical infections by *Fusarium* species are notoriously difficult to treat, due to delay in correct diagnosis, but particularly because of the intrinsic resistance to most currently used antifungal drugs (10). Voriconazole, amphotericin B, and posaconazole show some activity *in vitro* but exhibit limited effectiveness in clinical treatment (9). *In vitro* susceptibility is observed to lanoconazole and luliconazole (11), but these drugs are limited to topical application. Thus, for the most vulnerable, immunocompromised patient cohorts, antifungals with new modes of action are required for the management of fusariosis.

Olorofim (formerly F901318, developed by F2G, Inc., Manchester, UK) is a new antifungal drug, belonging to the orotomide class of antifungals. This agent is different from other antifungal drugs in that it interferes with pyrimidine synthesis by inhibiting dihydroorotate dehydrogenase, thus slowing down fungal growth (12). Olorofim is effective against many filamentous fungi and the thermally dimorphic fungi but lacks activity against yeasts and Mucorales (13). The role of olorofim in the treatment of fusariosis remains unclear due to variable *in vitro* activity reported in published studies (14–17). The recent phase 2b open-label trial of 204 patients with various invasive fungal infections with few or no alternative treatment options treated with olorofim included three patients with invasive fusariosis (18). Although the *Fusarium* isolates from these three patients showed low olorofim MICs, the outcome was not reported. The objective of the present study was to describe the *in vitro* susceptibility of well-characterized *Fusarium* isolates to olorofim in order to provide evidence for the future clinical application of this compound.

## MATERIALS AND METHODS

### Strains

**Fig 1:**
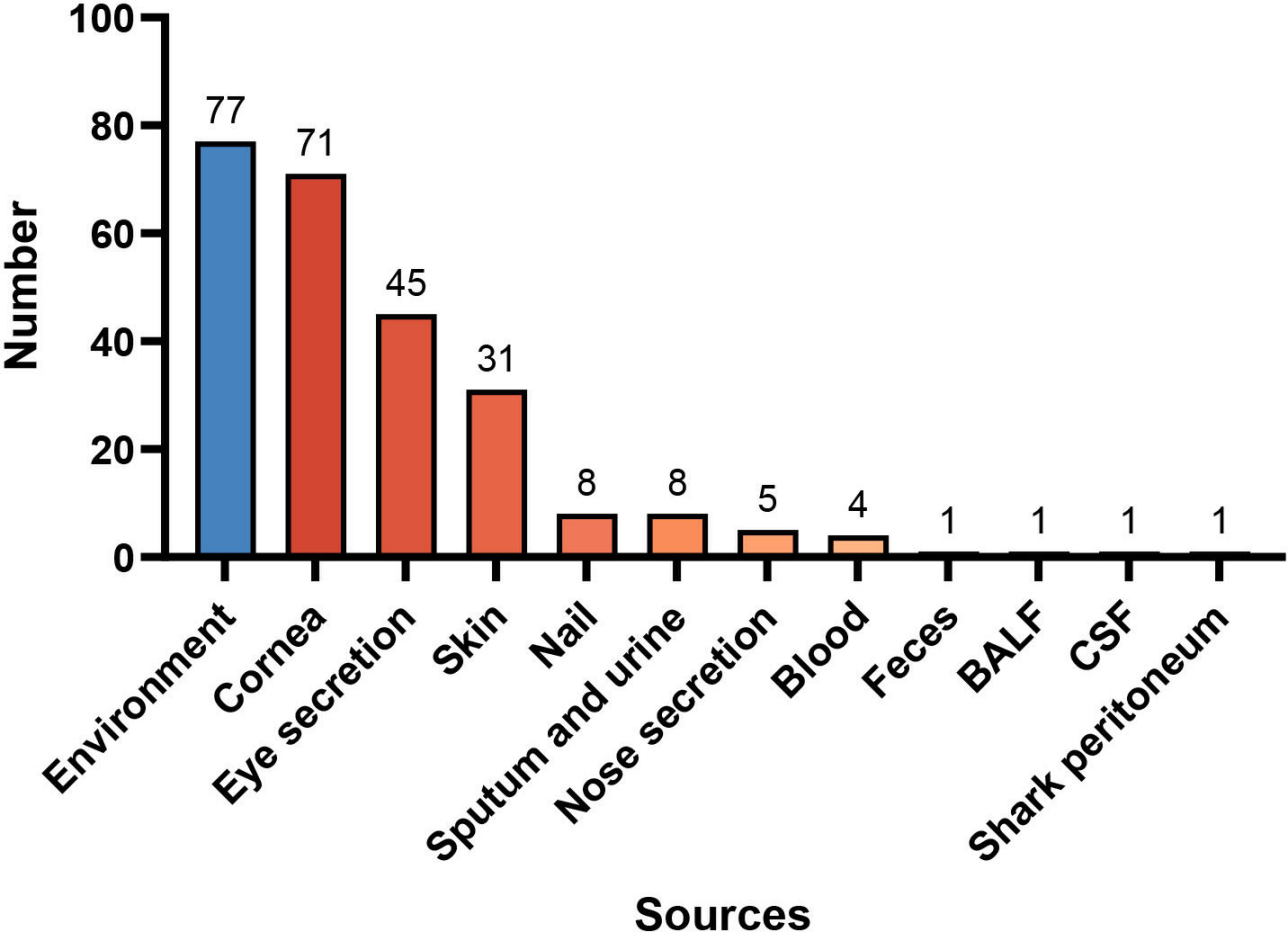
Different sources of 253 strains of *Fusarium* spp. BALF, bronchoalveolar lavage fluid; CSF, cerebro-spinal fluid.

The total of 253 isolates were cultured from patients (*n* = 176) and the environment (*n* = 77) since May 1995 and conserved in the reference culture collection of the Research Center for Medical Mycology at Peking University, Beijing, China. Clinical isolates were recovered from cornea tissue (28.1%, 71/253), eye secretion (17.8%, 45/253), skin tissue and secretion (12.3%, 31/253), nail (3.2%, 8/253), sputum and urine (3.2%, 8/253), nose secretion (2.0%, 5/253), blood (1.6%, 4/253), feces (0.4%, 1/253), bronchoalveolar lavage fluid (0.4%, 1/253), cerebro-spinal fluid (0.4%, 1/253), and shark peritoneum (0.4%, 1/253, non-human source), with all clinical isolates derived exclusively from human patients except the singular shark peritoneal specimen (Fig. 1). Environmental sources were plant tomato (*Solanum lycopersicum*), wheat (*Triticum aestivum*), cashew (*Anacardium occidentale*), carrot (*Daucus carota*), carnation (*Dianthus caryophyllus*), rice (*Oryza sativa*), maize (*Zea maydis*), wild oats (*Avena fatua*), barley (*Hordeum vulgare*), banana (*Musa acuminata*), asparagus (*Asparagus officinale*), lily (*Lilium longiflorum*), leaves of unknown species, soil, and some unknown sources in 30.4% (77/253). With the exception of the shark peritoneum isolate originating from Germany, all clinical strains were sourced from China, while environmental strains originated from El Salvador, France, Germany, Italy, the Netherlands, the Philippines, Spain, Uruguay, and the USA.

### Illumina sequencing

After strains recovery from storage, cultures were established on potato dextrose agar and incubated at 28°C for 72 h. Genomic DNA was isolated using the Fungal Genomic DNA Extraction Kit from BioFlux Biospin. Whole-genome sequencing was performed on the Illumina NovaSeq 6000 platform (Illumina Inc., USA) with 2 × 150 bp paired-end libraries (insert size: 350 bp), generating >100 × coverage depth per genome.

### Genome assembly

Raw sequencing reads were first processed using FLASH v1.2.11 to merge overlapping paired-end reads with parameters -m 10 × 0.2 M 250. Merged and unmerged reads were then quality-trimmed using Trimmomatic v0.39 with parameters SLIDINGWINDOW:4:20 LEADING:3 TRAILING:3 MINLEN:50. The filtered high-quality reads were *de novo* assembled using SPAdes v3.15.3 with the—isolate option optimized for isolate genomes. Assembly quality was assessed using BUSCO v5.5 with the hypocreales_odb10 data set to evaluate completeness, with contigs < 500 bp filtered post-assembly.

### Barcode gene extraction

Protein-coding genes were annotated using Funannotate v1.8.13 with transcripts evidence from clade hypocreales from Uniprot database. For phylogenetic analysis, three loci—*RPB1*, *RPB2*, and *TEF1*—were extracted by aligning reference sequences from the *Fusarium* MLST database (fusarium.org) against the annotated genes using BLASTN v2.13.0. Best-hit sequences with ≥90% query coverage and ≥80% identity were retained.

For gene locus *pyrE*, the reference protein-coding sequences of different species (including *F. proliferatum*, *F. solani*, *F.oxysporum*, *F. verticillioides*, *F. flagelliforme,* and *F. coffeatum*) were retrieved from NCBI nucleotide database and aligned with the annotated genes using BLASTN v2.13.0. Best-hit sequences with ≥90% query coverage and ≥80% identity were retained. Gene body and 1.5 kb flanking sequences of the best-hit gene locus were extracted, and the intron and flanking sequences were lowercase masked to distinguish with uppercased exon sequences. The DNA sequences were translated into protein sequences using the Expasy online Translate tool (https://web.expasy.org/translate/). The resulting protein sequences were then aligned using the SnapGene (v7.1.2). Subsequently, sequence alignment figures were generated using the ESPript 3.0 online server (https://espript.ibcp.fr/ESPript/ESPript/index.php).

### Strain identification

The strains were identified through a polyphasic approach combining genomic and phylogenetic analyses. For phylogenetic resolution, three protein-coding loci—*RPB1* (RNA polymerase II largest subunit), *RPB2* (RNA polymerase II second largest subunit), and *TEF1* (translation elongation factor 1-alpha)—were extracted from the assembled genomes. These loci were selected due to their established utility in resolving species-level relationships within *Fusarium* and related genera. Reference sequences of type strains and closely related species were retrieved from *Fusarium* MLST database (fusarium.org).

Multiple sequence alignments were generated and manual refinement in MAFFT (v7.487) with the --localpair --maxiterate 1000 parameters to optimize for homologous but distantly related sequences. The best-fit substitution models were determined using IQ-TREE (v2.0.3) built-in ModelTest function. Maximum Likelihood (ML) phylogenetic trees were reconstructed with IQ-TREE with parameters --alrt 1000 (for SH-aLRT branch tests) -B 1000 (incorporating 1,000 ultrafast bootstrap replicates to assess branch support) and -o Bisifusarium_aseptatum_CGMCC_3.20816_T, Bisifusarium_nectrioides_CBS_176.31_LT (to specify outgroup taxa). Bayesian Inference (BI) was optionally performed using MrBayes.

The final phylogenetic trees were visualized and annotated in iTOL, with bootstrap values ≥ 90% considered strongly supportive of clade stability. Strains were assigned to species based on their clustering with type strains or well-characterized reference sequences in the multilocus phylogeny.

### Antifungal agents and *in vitro* susceptibility

Strains were tested using the broth microdilution format according to CLSI M38, 3rd ed. reference method (19). Olorofim was obtained as reagent-grade powder provided by F2G Inc., UK, and was used in concentrations of 0.015–16 µg/mL. Olorofim stock solutions were prepared in dimethyl sulfoxide (DMSO) at 1,600 µg/mL and serially diluted in RPMI-1640 to achieve final test concentrations, with DMSO rigorously maintained at ≤1% (vol/vol) in all 200 µL assay wells. Strains were grown on potato dextrose agar for 48 h at 35°C and then until day 7 at 28°C to induce conidiation. Conidia were collected by gently flushing 5 mL phosphate buffer saline (pH 7.4) on colonies and aspirating the suspension into a sterile collection tube. Suspensions were counted on a hemocytometer and diluted in RPMI 1640 to the desired concentration of (0.4–5) × 10^4^ CFU/mL. Microdilution plates were incubated at 35°C for 48 h, and the MICs were defined as the lowest concentration with complete growth inhibition compared to the drug-free growth. Each antimicrobial susceptibility test was performed in triplicate. The final result was determined based on majority agreement, selecting the outcome shared by at least two of the three replicates.

### Statistical analysis

MIC_90_ were obtained by ordering the data for each antifungal in the ascending order and selecting the 90th quantile. Geometric mean (GM) MICs were calculated using Microsoft Office Excel 2019 software. The upper limit of wild-type (UL-WT) was calculated by the 2010 updated version of the ECOFFinder program which is available from the CLSI website (https://clsi.org/resources/ecoffinder/). Statistical analyses were conducted via GraphPad Prism (version 10.3.0). Kruskal-Wallis tests analyzed group differences for nonparametric data sets, followed by Bonferroni-adjusted pairwise tests. Outcomes are reported as [min, max] ranges (*P* < 0.05 significance). MICs exceeding the highest tested concentration were imputed as one dilution higher for statistical analyses.

## RESULTS

**Fig 2:**
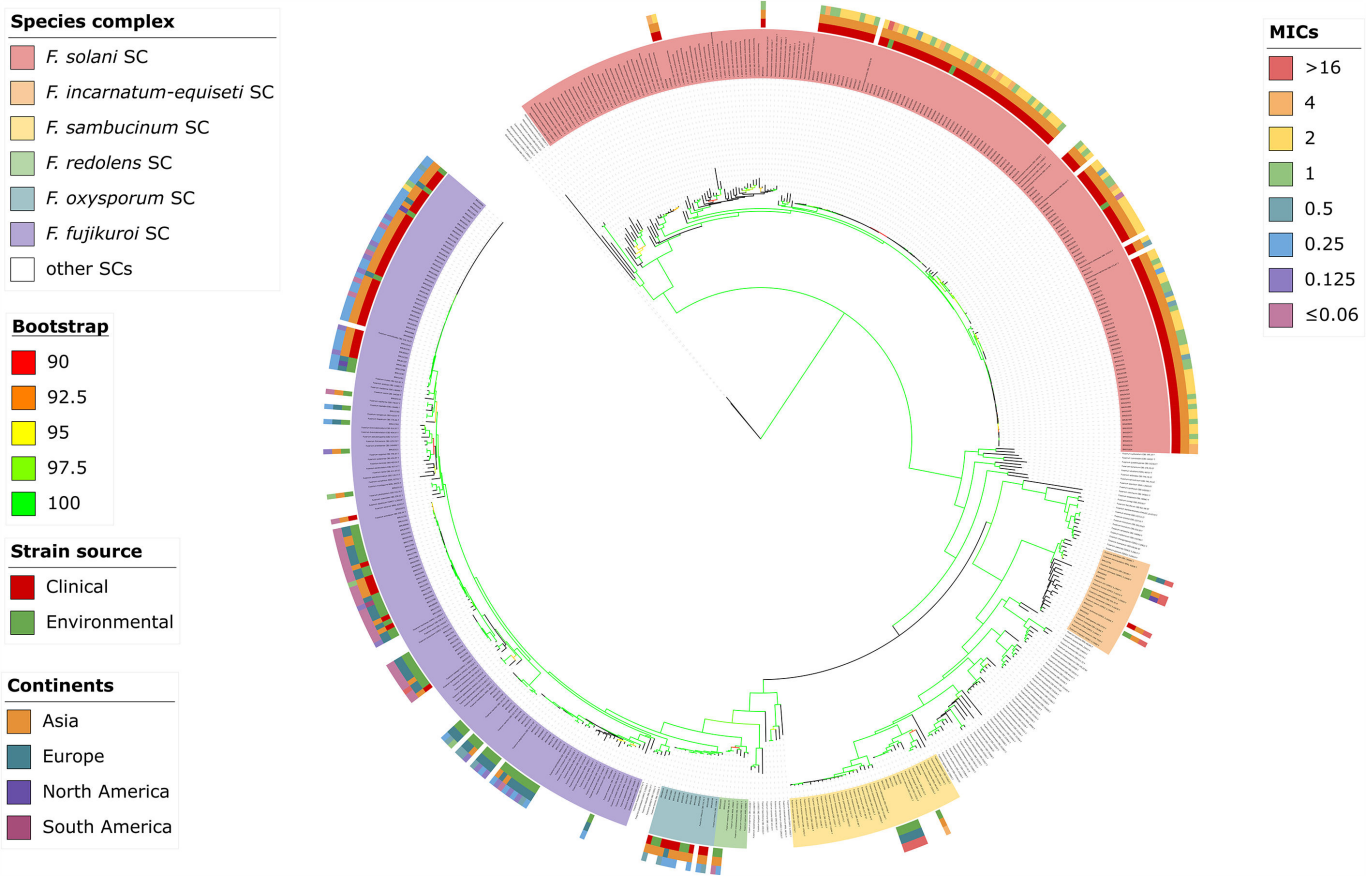
Phylogenetic tree of 253 strains of *Fusarium* spp. The diagram features three concentric outer rings, arranged from innermost to outermost as follows: strain origin (clinical strains or environmental strains), continent of isolation, and MIC distribution. Reference strains shown without color.

All 253 *Fusarium* isolates were identified based on multiple target genes (Fig. 2) and were assigned to six SCs, as follows: FSSC (syn. *Neocosmospora*, 48.2%, *n* = 122), *F. fujikuroi* SC (41.9%, *n* = 106), FOSC (4.7%, *n* = 12), *F. sambucinum* SC (2.4%, *n* = 6), *F. incarnatum-equiseti* SC (2.0%, *n* = 5), and *F. redolens* SC (0.8%, *n* = 2). Within FSSC, the following named sibling species were included: *F. solani s.str*. (*n* = 54), *F. falciforme* (*n* = 43), *F. keratoplasticum* (*n* = 21), *F. metavorans* (*n* = 2), *F. amplum* (*n* = 1), and *F. suttonianum* (*n* = 1); within *F. fujikuroi* SC: *F. verticillioides* (*n* = 45), *F. annulatum* (*n* = 26)*, F. subglutinans* (*n* = 12), *F. elaeagni* (*n* = 9), *F. sacchari* (*n* = 7), *F. brevicatenulatum* (*n* = 1), *F. konzum* (*n* = 1), *F. napiforme* (*n* = 1), *F. nygamai* (*n* = 1), *F. phyllophilum* (*n* = 1), *F. musae* (*n* = 1), and *F. ramigenum* (*n* = 1) (Table 1).

**TABLE 1:** Distribution of olorofim MICs against *Fusarium* species^*a*^

Species complex	Species (count)	Distribution of MIC (μg/mL)												MIC_90_ (μg/mL)	GM
		0.015	0.03	0.06	0.125	0.25	0.5	1	2	4	8	16	>16		
*F. solani* SC	(122)			1		1	6	34	68	11			1	2	1.602
	*F. solani* (54)						1	16	29	7			1	4	1.828
	*F. falciforme* (43)					1	4	13	23	2				2	1.403
	*F. keratoplasticum* (21)			1			1	4	14	1				2	1.435
	*F. metavorans* (2)								1	1				4	2.828
	*F. amplum* (1)							1						1	1
	*F. suttonianum* (1)								1					2	2
*F. fujikuroi* SC	(106)	10	6	21	15	41	7	4	1				1	0.5	0.137
	*F. verticillioides* (45)		4		6	31	2	1	1					0.25	0.210
	*F. annulatum* (26)	3	2	18	2			1						0.125	0.057
	*F. subglutinans* (12)			1	5	5	1							0.25	0.176
	*F. elaeagni* (9)	7		1									1	>16	0.041
	*F. sacchari* (7)				1	1	4	1						1	0.410
	*F. brevicatenulatum* (1)					1								0.25	0.25
	*F. napiforme* (1)			1										0.06	0.06
	*F. nygamai* (1)				1									0.125	0.125
	*F. phyllophilum* (1)							1						1	1
	*F. ramigenum* (1)					1								0.25	0.25
	*F. konzum* (1)					1								0.25	0.25
	*F. musae* (1)					1								0.25	0.25
*F. oxysporum* SC	*F. inflexum* (12)				1	8	3							0.5	0.281
*F. sambucinum* SC	(6)									1			5	>16	22.627
	*F. sibiricum* (5)												5	>16	32
	*F. parabolicum* (1)									1				4	4
*F. incarnatum-* *equiseti* SC	(5)												5	>16	32
	*F. ipomoeae* (1)												1	>16	32
	*F. nanum* (1)												1	>16	32
	*F. guilinense* (1)												1	>16	32
	*F. citri* (1)												1	>16	32
	*F. lacertarum* (1)												1	>16	32
*F. redolens* SC	*F. redolens* (2)		1			1								0.25	0.087

^*a*^ MIC90: MIC at which 90 % of isolates are inhibited; GM, geometric mean; SC, species complex.

A total of 253 isolates were tested for *in vitro* antifungal susceptibility against olorofim; distribution of MIC values is shown in Table 1. The GM of the MICs of olorofim for all 253 isolates was 0.581 µg/mL, ranging from 0.087 µg/mL for *F. redolens* SC to 32 µg/mL for *F. incarnatum-equiseti* SC. The FSSC and *F. fujikuroi* SC were the most prevalent SCs encountered in the hospital and included in our study. A significant difference in susceptibility was observed between FSSC (GM MIC 1.602 µg/mL) and *F. fujikuroi* SC (GM MIC 0.137 µg/mL) (*P* < 0.05). GM values indicate moderate variation across SCs. All *F. sibiricum* and *F. incarnatum-equiseti* SC strains showed high MICs of olorofim, with MIC values exceeding 16 µg/mL. Overall, significant interspecies variability was observed: GMs ranged from 1.403 to 2.828 µg/mL for FSSC and from 0.06 to 1 µg/mL for *F. fujikuroi* SC. Intra-species variability in species with >10 isolates tested ranged from 0.06 to >16 in *F. solani s. str*., defined as the pathotype containing the type strain of *F. solani* (20).

We also determined the upper limits of wild-type (WT) MIC (UL-WT) based on the 97.5% and 99% MICs (Table 2). A 97.5% UL-WT and 99% UL-WT of *F. solani* were observed for 4 µg/mL with 12.9% of the isolates above the UL-WT. Meanwhile, a 97.5% UL-WT and 99% UL-WT of *F. verticillioides* were calculated at 0.5 µg/mL, and the percentage of isolates above the UL-WT was 7.26%.

**TABLE 2:** MIC and UL-WT distribution of *Fusarium* isolates against olorofim^*a*^

Species	No. of isolates	Range (μg/mL)	Modal MIC	UL-WT 97.5%	UL-WT 99%	% NWT 97.5%	% NWT 99%
*F. solani*	54	0.5–> 16	2	4	4	12.9	12.9
*F.verticillioides*	45	0.03–2	0.25	0.5	0.5	7.26	7.26

^*a*^ Modal MIC, the most frequently obtained MIC. WT, wild-type; NWT, non-wild-type; UL-WT, upper limit of wild-type MIC.

**Fig 3:**
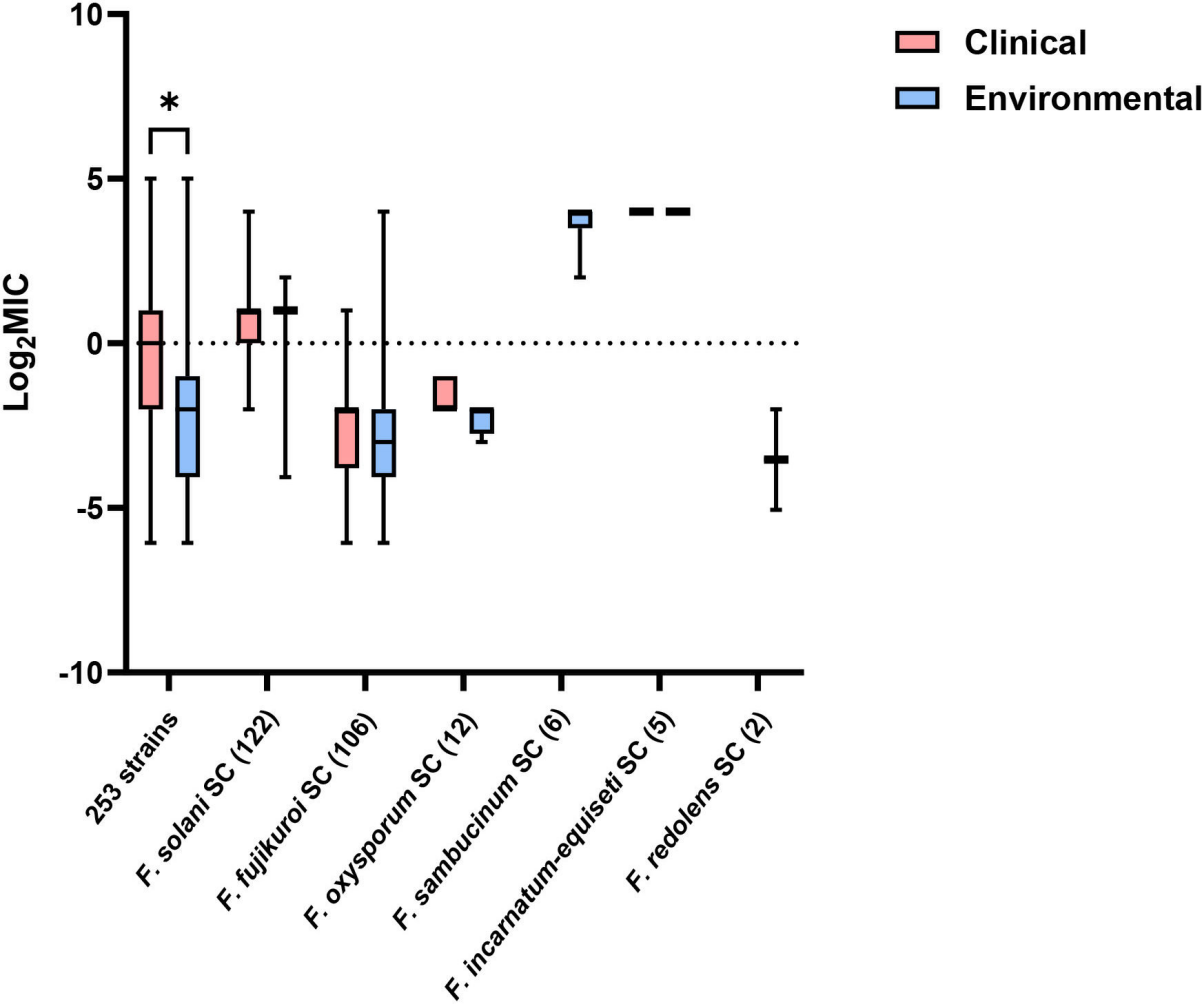
MIC comparison between clinical-origin and environmental-origin strains. Numbers in parentheses denote isolate counts. * indicates *P* < 0.05.

The 253 fungal strains were categorized into clinical-origin and environmental-origin groups for MIC distribution comparison. The clinical-origin group exhibited significantly higher MIC values than the environmental-origin group (*P <* 0.05). However, this phenomenon was not observed across different species complexes (Fig. 3).

**Fig 4:**
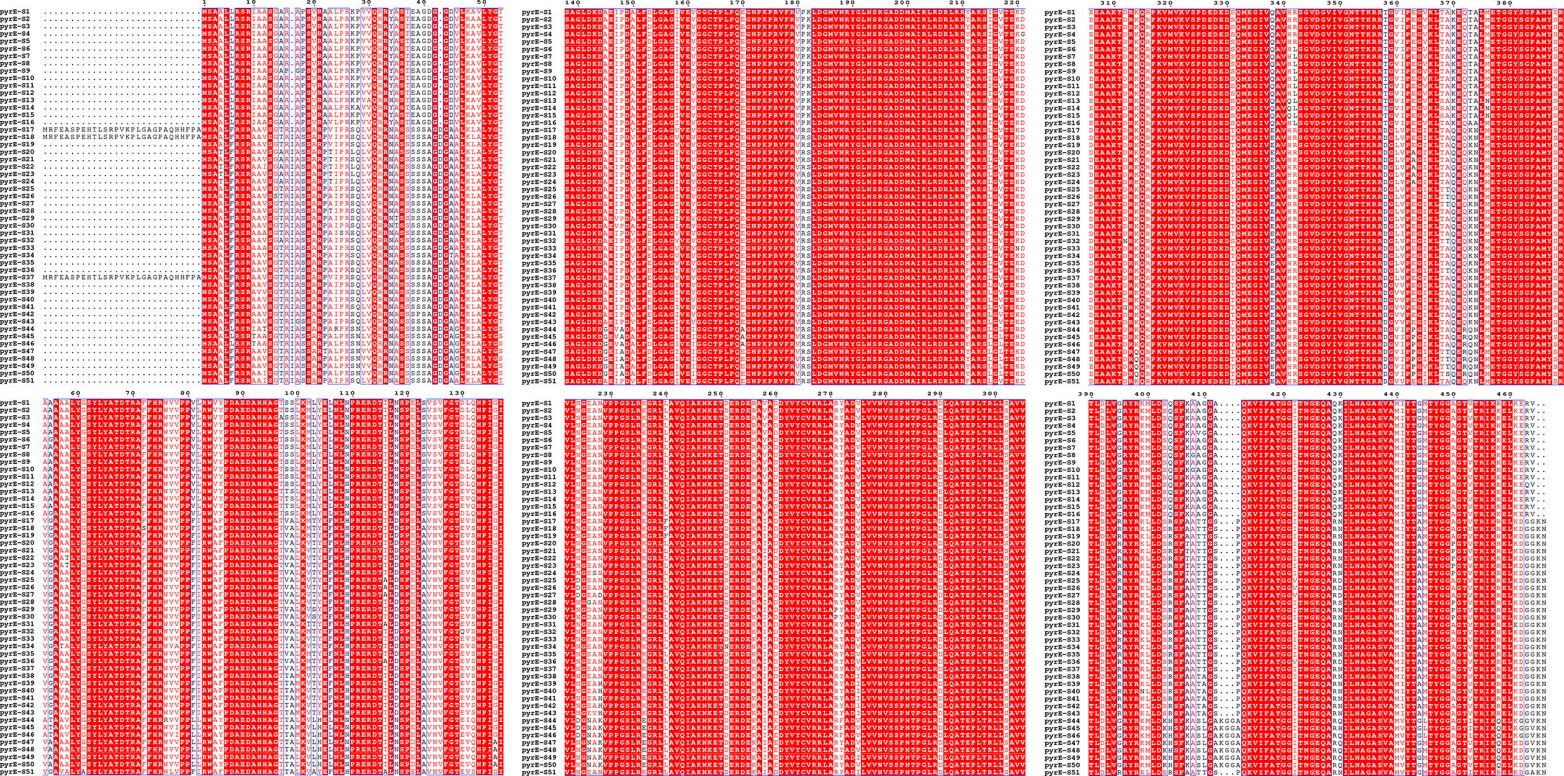
Alterations of pyrE amino acid sequence in *Fusarium* spp. Amino acid diversity of 51 types of pyrE alterations.

To explore the association in *Fusarium* and *pyrE* genes, we aligned the pyrE proteins. Detailed analysis of the translated pyrE protein sequences revealed substantial diversity: translation of the 253 gene sequences resulted in 51 distinct protein subtypes (Fig. 4). This high level of sequence variation underscores the genetic plasticity of pyrE protein within the studied *Fusarium* isolates. Most subtypes were represented by only a single isolate; the predominant subtypes were pyrE-S1 (*n* = 49 isolates), pyrE-S17 (*n* = 42), and pyrE-S6 (*n* = 22) (Table 3).

**TABLE 3:** The 51 pyrE protein subtypes: isolate count, species, and MIC range

Substitutions	Count	Species complex	Species	MIC range (μg/mL)
S1	49	*F. solani SC*	*Fusarium solani*	0.5–4
S2	1	*F. solani SC*	*Fusarium solani*	4
S3	1	*F. solani SC*	*Fusarium solani*	>16
S4	1	*F. solani SC*	*Fusarium solani*	2
S5	2	*F. solani SC*	*Fusarium solani*	1–4
S6	22	*F. solani SC*	*Fusarium falciforme*	0.25–4
S7	11	*F. solani SC*	*Fusarium falciforme*	0.5–2
S8	8	*F. solani SC*	*Fusarium falciforme*	0.5–2
S9	1	*F. solani SC*	*Fusarium falciforme*	1
S10	1	*F. solani SC*	*Fusarium falciforme*	2
S11	18	*F. solani SC*	*Fusarium keratoplasticum*	0.06–4
S12	3	*F. solani SC*	*Fusarium keratoplasticum*	1–2
S13	1	*F. solani SC*	*Fusarium metavorans*	2
S14	1	*F. solani SC*	*Fusarium metavorans*	4
S15	1	*F. solani SC*	*Fusarium amplum*	1
S16	1	*F. solani SC*	*Fusarium suttonianum*	2
S17	42	*F. fujikuroi SC*	*Fusarium verticillioides*	0.03–2
S18	1	*F. fujikuroi SC*	*Fusarium verticillioides*	0.125
S19	2	*F. fujikuroi SC*	*Fusarium verticillioides*	0.25
S20	16	*F. fujikuroi SC*	*Fusarium annulatum*	0.015–1
S21	5	*F. fujikuroi SC*	*Fusarium annulatum*	0.06–0.125
S22	1	*F. fujikuroi SC*	*Fusarium annulatum*	0.06
S23	1	*F. fujikuroi SC*	*Fusarium annulatum*	0.125
S24	3	*F. fujikuroi SC*	*Fusarium annulatum*	0.06
S24	1	*F. incarnatum-equiseti SC*	*Fusarium citri*	>16
S25	7	*F. fujikuroi SC*	*Fusarium subglutinans*	0.06–0.25
S26	4	*F. fujikuroi SC*	*Fusarium subglutinans*	0.125–0.25
S27	1	*F. fujikuroi SC*	*Fusarium subglutinans*	0.5
S28	9	*F. fujikuroi SC*	*Fusarium elaeagni*	0.015–>16
S29	4	*F. fujikuroi SC*	*Fusarium sacchari*	0.125–1
S30	3	*F. fujikuroi SC*	*Fusarium sacchari*	0.5
S31	1	*F. fujikuroi SC*	*Fusarium brevicatenulatum*	0.25
S32	1	*F. fujikuroi SC*	*Fusarium napiforme*	0.06
S33	1	*F. fujikuroi SC*	*Fusarium nygamai*	0.125
S34	1	*F. fujikuroi SC*	*Fusarium phyllophilum*	1
S35	1	*F. fujikuroi SC*	*Fusarium ramigenum*	0.25
S36	1	*F. fujikuroi SC*	*Fusarium konzum*	0.25
S37	1	*F. fujikuroi SC*	*Fusarium musae*	0.25
S38	4	*F. oxysporum SC*	*Fusarium inflexum*	0.125–0.5
S39	2	*F. oxysporum SC*	*Fusarium inflexum*	0.25
S40	2	*F. oxysporum SC*	*Fusarium inflexum*	0.25
S41	2	*F. oxysporum SC*	*Fusarium inflexum*	0.25
S42	1	*F. oxysporum SC*	*Fusarium inflexum*	0.25
S43	1	*F. oxysporum SC*	*Fusarium inflexum*	0.5
S44	1	*F. sambucinum SC*	*Fusarium parabolicum*	4
S45	4	*F. sambucinum SC*	*Fusarium sibiricum*	>16
S46	1	*F. sambucinum SC*	*Fusarium sibiricum*	>16
S47	1	*F. incarnatum-equiseti SC*	*Fusarium nanum*	>16
S48	1	*F. incarnatum-equiseti SC*	*Fusarium guilinense*	>16
S49	1	*F. incarnatum-equiseti SC*	*Fusarium lacertarum*	>16
S50	1	*F. incarnatum-equiseti SC*	*Fusarium ipomoeae*	>16
S51	2	*F. redolens SC*	*Fusarium redolens*	0.03–0.25

Overall, no significant mutations were identified in the pyrE sequences of non-WT *Fusarium* isolates compared to WT counterparts, with the exception of the pyrE-S3 subtype. Isolates of the pyrE-S3 subtype exhibited MICs > 16 µg/mL and harbored a unique T97A substitution not observed in other subtypes. Furthermore, isolates sharing the same protein subtype exhibited a wide range of MIC values; for instance, the MIC range for isolates of the pyrE-S28 subtype spanned from 0.015 µg/mL to >16 µg/mL. Conversely, isolates with identical MIC values frequently possessed different pyrE protein subtypes, even when belonging to the same *Fusarium* species.

## DISCUSSION

The treatment of fusarioses remains challenging due to the intrinsic multidrug resistance of *Fusarium* species. The drugs with potential systemic administration that have proven to be more effective against fusariosis are voriconazole, amphotericin B, and posaconazole (2, 21). An international consortium of 17 laboratories determined epidemiological cutoff values (ECVs) for *Fusarium* species according to the CLSI guidelines. The MIC distributions were as follows: for amphotericin B 4–8 µg/mL, posaconazole 2–32 µg/mL, and voriconazole 4–32 μg/mL (22). As yet, no clinical breakpoints (CBPs) have been defined for *Fusarium* species, and most currently used antifungal drugs show poor *in vitro* activity (9, 10). However, in general, the clinical response to *Fusarium* infections was considered to be modest, and the relevance of MICs for clinical outcome remained unclear (23, 24).

Olorofim is a novel orotomide class of antifungals which has a broad spectrum of activity against molds, with the exception of Mucorales (16). The compound demonstrated *in vitro* activity against important agents of systemic mycoses such as *Coccidioides immitis* and *C. posadasii* (25). *In vitro* susceptibility of *Fusarium* to olorofim was considered to be variable. Olorofim drug susceptibility testing conducted by Jørgensen et al. (17) indicated that the MIC of *F. dimerum* (*n* = 1) and siblings of the FSSC (*n* = 8) was >1 µg/mL, while that of *F. proliferatum* (*n* = 1) was 0.06 µg /mL, reflecting some species-specific variation. Georgacopoulos et al. (14) showed no antifungal activity of olorofim against *F. chlamydosporum*, *F. dimerum,* and FSSC (MIC range 2 to >2 µg/mL), and a wide range of antifungal activity against *F. oxysporum* (MIC 0.12 to >2 µg/mL). *Fusarium moniliforme* (an outdated name for the later split species *F. proliferatum* and *F. verticillioides*) and *F. verticilloides* (both members of the *F. fujikuroi* SC) had low MICs compared to remaining species of the genus. In contrast, Badali et al. (15) observed good *in vitro* activity of olorofim against members of FOSC (*n* = 45) and FSSC (*n* = 16). Against FOSC isolates, olorofim MICs ranged between 0.03–0.5 µg/mL and 0.06−>4 µg/mL at the 50% and 100% inhibition endpoints, respectively. Against FSSC isolates, olorofim MIC ranged between 0.25–1 µg/mL and 1−>4 µg/mL at 50% and 100% inhibition, respectively. Nevertheless, as noted by others, the olorofim MIC values against *Fusarium* were relatively low (GM of the MICs = 0.792 µg/mL) when compared to most other antifungals (14, 26, 27).

The effect of olorofim against *Fusarium* species seems to differ between species complexes. Our data indicate that the FSSC is significantly more resistant to olorofim (GM MIC = 1.602 µg/mL) than the *F. fujikuroi* SC (GM MIC = 0.137 µg/mL). Notably, several authors maintain that the FSSC deserves a separate position as the genus *Neocosmospora* on the basis of phylogeny (28). It may be concluded that the *Fusarium* SCs respond differentially to olorofim, but an association of resistance and phylogeny remains unclear. This question needs a larger number of strains of clinically less-known SCs to be investigated to obtain statistic support for the differences observed in Table 1.

In general, variation between strains was observed, notably in *F. fujikuroi* SC (MIC 0.015−>16 µg/mL) and FSSC (MIC 0.06−>16 µg/mL), with individual strains deviating by highest degrees of MIC, which suggests that accurate species identification does not predict susceptibility to olorofim.

We observed that while MIC₉₀ and GM values generally exhibited concordant directional trends across most strains, GM provides superior resolution of intra-species MIC clustering when MIC₉₀ values are identical. This is exemplified by *F. fujikuroi* SC and *F. oxysporum* SC, both sharing MIC₉₀ 0.5 µg/mL, where the lower GM for *F. fujikuroi* SC (0.137 µg/mL vs 0.281 µg/mL in *F. oxysporum* SC) indicates tighter central tendency clustering toward lower concentrations.

We calculated the UL-WT only based on the value of MIC in order to better describe the sensitivity of olorofim to *Fusarium*. However, a low MICs do not necessarily correlate with clinical efficacy. To comprehensively evaluate therapeutic outcomes, preclinical validation through animal models is required.

During the evaluation of *Fusarium* susceptibility to olorofim, notable strain-specific discrepancies were observed (29). A single isolate of *F. solani* exhibited a MIC >16 µg/mL, contrasting with the majority of isolates (MIC ≤ 4 µg/mL). Similarly, one *F. elaeagni* strain demonstrated exceptionally high MIC > 16 µg/mL, while all other isolates showed low MICs (MIC ≤ 0.06 µg/mL). These outliers suggest potential acquired resistance mechanisms distinct from the general population or may indicate taxonomic variability.

The observed MIC elevation in clinical strains compared to environmental strains is probably confounded by the imbalanced distribution of clinical and environmental isolates within SCs. Notably, FSSC strains dominated the data set, with all but one isolate originating from clinical sources and demonstrating characteristically high MIC values. This suggests that the apparent MIC elevation in clinical strains might reflect the intrinsically higher MIC levels of FSSC rather than true source-dependent differences. In contrast, *F. fujikuroi* SC, which contained more balanced numbers of clinical and environmental isolates, showed no significant MIC differences between sources. Based on the current data, conclusive interpretation of MIC differences between strain origins remains elusive. Further analysis with larger collections of diverse-origin isolates is required to clarify this relationship.

Previous studies in *Aspergillus* species have linked olorofim resistance to mutations in the *pyrE* gene (encoding dihydroorotate dehydrogenase) (30). While substantial sequence variation exists within the pyrE protein across studied *Fusarium* isolates, our data reveal that the majority of amino acid substitutions show no discernible correlation with olorofim MIC. The T97A substitution was uniquely associated with high MIC (>16 µg/mL) in isolates of the pyrE-S3 subtype; however, this specific substitution was not observed in other non-WT isolates harboring different pyrE subtypes.

Although the T97A substitution may potentially contribute to altered olorofim susceptibility in the specific genetic context of the pyrE-S3 subtype, its isolated occurrence and lack of recurrence in other non-WT strains provide limited evidence for a generalizable role in *Fusarium* olorofim phenotype. Collectively, these findings indicate that reduced susceptibility to olorofim in *Fusarium* is more likely governed by mechanisms independent of *pyrE*-mediated pathways. Potential alternative mechanisms could include efflux pump activation, compensatory metabolic adaptations, alterations in additional drug targets, or epigenetic regulation. Further investigations are warranted to elucidate the polygenic or epigenetic factors governing olorofim susceptibility in *Fusarium*.

In conclusion, even though the activity of olorofim against *Fusarium* species was moderate and variable, the compound may be a useful addition to the panel of antifungals to be used against multi-drug resistant agents of fusarioses. However, low olorofim MIC may not predict clinical efficacy, and animal experiments are critical to further characterize clinical efficacy. Since olorofim offers a novel mechanism of action, its contribution to eradication of the etiologic agent may be promising as a supplementary drug against invasive *Fusarium* infections. More studies are needed to explain and establish the potential use of the orotomide class in interacting with the extensive inter-species diversity of the genus *Fusarium* and the correlation between *in vitro* and *in vivo* susceptibilities.

## Data Availability

The nucleotide sequences of the RPB1, RPB2, TEF1, and pyrE gene fragments from the strains studied in this work have been deposited in the Zenodo database using the DOI 10.5281/zenodo.16891147.
